# Outbreak-Associated Novel Avipoxvirus in Domestic Mallard Ducks, China

**DOI:** 10.3201/eid2102.140215

**Published:** 2015-02

**Authors:** Min Zheng, Huihui Cao, Xiankai Wei, Yong Qin, Shaoyi Ou, Baoxue Huang, Mingguo He, Zhiping Xia, Liefeng Zheng, Jun Li, Qi Liu

**Affiliations:** Guangxi Center for Animal Disease Control and Prevention, Nanning, China (M. Zheng, X. Wei, L. Zheng, J. Li, Q. Liu);; Guangxi University, Nanning (H. Cao, X. Wei, Q. Liu);; Baise Center for Animal Disease Control, Baise, China (Y. Qin);; Daxin Center for Animal Disease Control, Daxin, China (S. Ou);; Tiandong Center for Animal Disease Control, Tiandong, China (B. Huang);; Xilin Center for Animal Disease Control, Xilin, China (M. He);; Academy of Military Medical Sciences, Changchun, China (Z. Xia)

**Keywords:** Avipoxvirus, duck, outbreak, China, viruses, mallard

**To the Editor:** In December 2013, an unidentified disease in domestic mallard ducks (*Anas platyrhynchos*) occurred in Guangxi Province, China. Rates of illness in adult male and female ducks were 50%–70% and 5%–30%, respectively, in different flocks. No deaths were observed. Clinical signs included cutaneous nodules on the birds’ eyelids, beaks, and legs. All classical endemic viruses and bacteria, including avian influenza virus, avian paramyxovirus 1, duck enteritis virus, *Riemerella anatipestifer*, and *Escherichia coli*, were excluded as causative agents by PCR or bacteria isolation. During investigation of the illness, we isolated a novel duck-pathogenic avipoxvirus (APV) from skin nodules of the affected ducks.

APVs, which contain a double-stranded DNA genome, are members of the genus *Avipoxvirus*, family *Poxviridae* (*1*). They naturally infect 232 species in 23 orders of wild and domestic birds worldwide (*2*). APV infections have 2 forms, cutaneous and diphtheritic, and can occur in either form or both forms. Cutaneous APV infection is characterized by nodular lesions on featherless areas of the body; diphtheritic APV infection usually results in higher death rates and produces nodular lesions on the mucous membranes of the mouth, esophagus, and/or trachea (*1*). In China, APV infections have been found in chickens, pigeons, turkeys, quail, and geese (*3*) but not in domestic ducks. APV antibodies were found once in a wild mallard duck (*4*), but no further etiologic and histopathologic evidence was found.

During December 2013–January 2014, farmers in 3 counties (Daxin, Xilin, and Tiandong; distance between each county 70–230 km) of Guangxi Province almost simultaneously reported to local veterinary services an unidentified disease in domestic mallard ducks. A total of 19 farms where domestic mallard ducks were raised (15 in Tiandong, 3 in Daxin, and 1 in Xilin) and 50,000 birds were affected. Affected ducks behaved and ate normally. Nodular lesions with scabs appeared on the unilateral or bilateral eyelids of affected ducks and on featherless regions on beaks and/or legs of 10% of affected ducks in some flocks (Technical Appendix Figure 1). No lesions were found in the digestive and respiratory tracts of affected ducks. Histopathologic examination of skin nodules using hematoxylin and eosin stain showed proliferative and necrotic dermatitis, with ballooning degeneration of keratinocytes, and large, eosinophilic ring-shaped Bollinger bodies (Technical Appendix Figure 2). Brick-shaped 330 × 280 × 200–nm virus particles with irregular pipe-shaped surface structures (Technical Appendix Figure 3), consistent with those of members of genus *Avipoxvirus*, were observed by electron microscopy. Ultrastructurall examination revealed that cytoplasm within degenerating epithelium contained inclusions comprising viral particles. The particles had a dumbbell-shaped central core, lateral bodies, and a convoluted outer membrane (Technical Appendix Figures 4, 5). Virus isolation from skin nodules was conducted on duck chorioallantoic membranes of specific pathogen–free embryonated 11-day-old duck eggs by using the method described by Joklik (*5*). At 7 days after infection, small pocks in chorioallantoic membranes and membrane thickening were observed. Three isolates from Xilin, Daxin, and Tiandong were designated as APV-XL, APV-DX, and APV-TD, respectively.

We amplified the partial sequences of *P4b* gene (*fpv167* locus) and DNA polymerase gene (*fpv094* locus) of the aforementioned 3 isolates using the specific primer pairs (P1: 5′-CAGCAGGTGCTAAACAACAA-3′, and P2: 5′-CGGTAGCTTAACGCCGAATA-3′ for P4b; PPolF: 5′-GGCYAGTACKCTTATYAAAGG-3′, and PPolR: 5′-CGTCTCTACGTGTTTCGCT-3′ for polymerase gene) (*6**,**7*). The sequences obtained (GenBank accession nos. KJ192189–KJ192191 for P4b gene, KM281932–KM281934 for polymerase gene) were aligned with published APV sequences. Among them, sequences from 3 Guangxi isolates were 100% nt identical to each other, suggesting they are the same virus. We further determined phylogenetic relationship using the Bayesian approach based on a general time reversible model with a γ distribution (GTR+G) implemented in MrBayes 3.2, as described by Gyuranecz et al. (*7*) and Ronquist et al. (*8*). The phylogenetic tree based on the concatenated sequences (981 bp, n = 62) of partial P4b (426 bp) and polymerase gene (555 bp) showed that Guangxi isolates group into a new cluster within subclade A5, which comprises viruses isolated from wild waterfowl, including mallard ducks, trumpeter swans, mottled ducks, redhead ducks, and blue-winged teal from North America (*6**,**7**,**9*). Estimated with MEGA according to the methods of Gyuranecz et al. (*7*) and Tamura et al. (*10*), we found that mean genetic distances of P4b genes, polymerase genes, and the concatenated sequences, between North American and Guangxi isolates were 0.002 ± 0.002 SE, 0.034 ± 0.004 SE, and 0.019 ± 0.003 SE, respectively (Figure).

**Figure:**
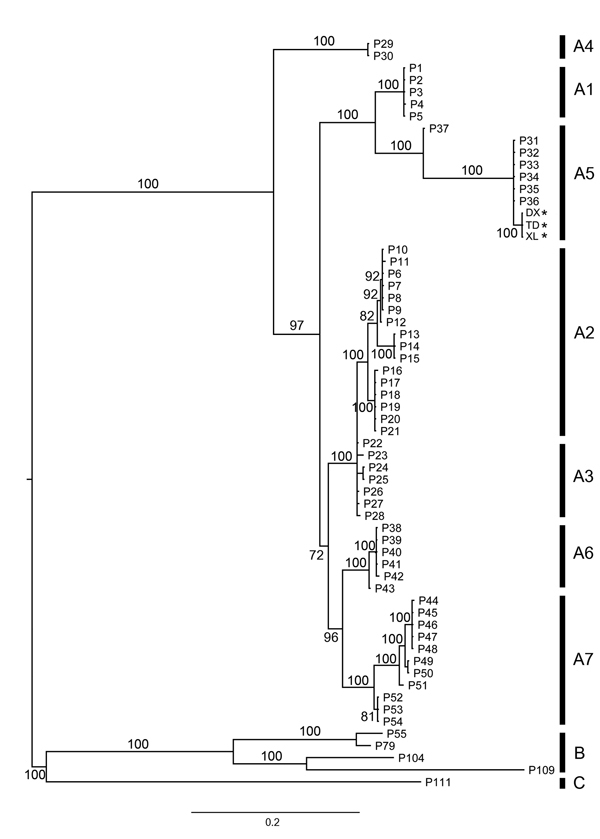
Bayesian phylogeny of concatenated DNA sequences (981 bp, n = 62) from genes encoding 4b core and DNA polymerase proteins of avipoxviruses. Posterior probability values of the Bayesian trees (1,000 replicates) are indicated. Asterisks (*) indicate sequences obtained in this. Avipoxvirus clades A–C and subclades are labeled according to the nomenclature of Jarmin et al. (*6*) and Gyuranecz et al. (*7*). Reference sequences used in this analysis are chosen from the study of Gyuranecz et al. (*7*). Scale bar indicates nucleotide substitutions per site.

Our findings highlight the possibility that APV has been recently introduced by wild waterfowl in the Northern Hemisphere into domestic mallard ducks. Further study is needed to determine the pathogenicity of this virus on other commercial poultry species and its influence on the poultry industry and wildlife protection.

Technical Appendix Figures showing cutaneous nodules on the eyelid and beak of a novel avipoxvirus–affected female mallard duck, China; proliferation of swollen keratinocytes in the epidermis; and characteristics of virus particles.
