# Supplementary material for: Outbreak-Associated Novel Avipoxvirus in Domestic Mallard Ducks, China
Source: Emerg Infect Dis. 2015 Feb;21(2):372–3. doi: 10.3201/eid2102.140215 (PMC4313631; doi:10.3201/eid2102.140215)
Supplement: Technical Appendix — Figures showing cutaneous nodules on the eyelid and beak of a novel avipoxvirus–affected female mallard duck, China; proliferation of swollen keratinocytes in the epidermis; and characteristics of virus particles. [file 14-0215-Techapp-s1.pdf]

Outbreak-associated Novel Avipoxvirus in Domestic Mallard Duck, China

Technical Appendix

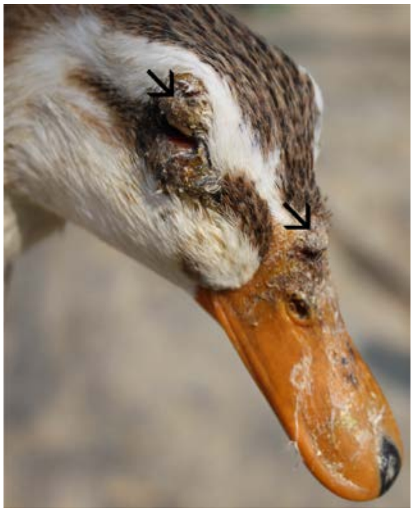

Technical Appendix Figure 1. Cutaneous nodules on the eyelid (arrow) and beak (arrow) of a novel avipoxvirus–affected female mallard duck, China

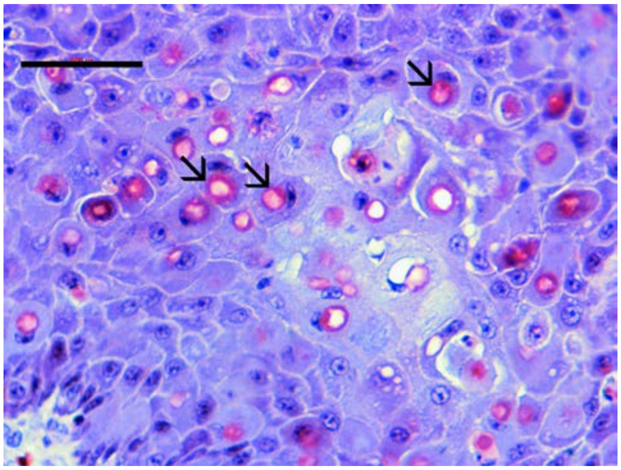

Technical Appendix Figure 2. Histologically, proliferation of swollen keratinocytes was observed in the epidermis. Degenerated cells included eosinophilic ring-shaped cytoplasmic inclusions (arrow), Bollinger bodies. Original magnification ×40, hematoxylin and eosin stain. Scale bar = 125 μm.

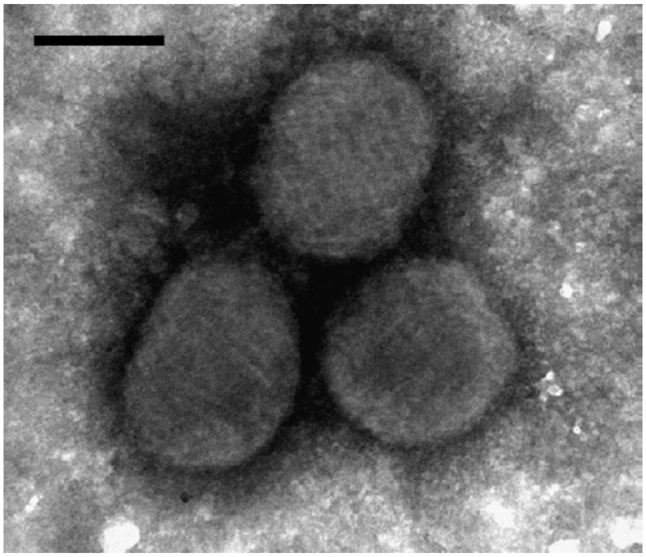

Technical Appendix Figure 3. Brick-shaped, 330 × 280 × 200–nm virus particles with irregular pipes-shaped surface structures. Original magnification ×100,000. Scale bar = 200 nm.

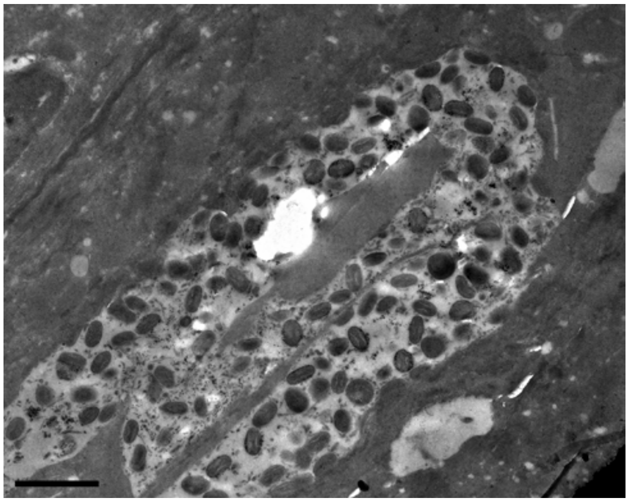

Technical Appendix Figure 4. Ultrastructurally, cytoplasm within degenerating epithelium contained inclusions that consisted of viral particles. Original magnification ×40,000. Scale bar = 1,000 nm.

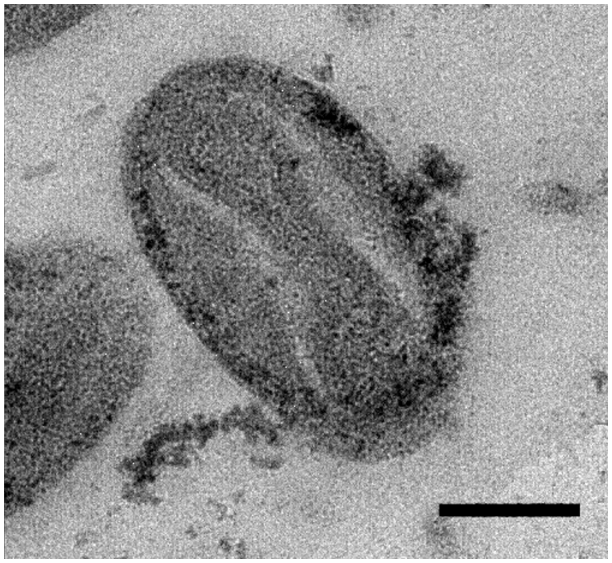

Technical Appendix Figure 5. Virus particle consists of a dumbbell-shaped central core, lateral bodies, and a convoluted outer membrane. Original magnification ×200,000. Scale bar = 100 nm.
